# The Synthesis of a Coumarin Carbohydrazide Dinuclear Copper Complex Based Fluorescence Probe and Its Detection of Thiols

**DOI:** 10.1371/journal.pone.0148026

**Published:** 2016-02-12

**Authors:** Guangjie He, Jing Li, Lu Yang, Chunhua Hou, Tianjun Ni, Zhijun Yang, Xinlai Qian, Changzheng Li

**Affiliations:** 1 Department of Forensic Medicine, Xinxiang Medical University, Jinsui Road No. 601, Xinxiang, 453003, P. R. China; 2 School of Basic Medical Science, Xinxiang Medical University, Jinsui Road No. 601, Xinxiang, 453003, P. R. China; 3 College of Pharmacy, Xinxiang Medical University, Jinsui Road No. 601, Xinxiang, 453003, P. R. China; University of Helsinki, FINLAND

## Abstract

Small-molecule thiols, such as cysteine (CYS) and glutathione (GSH), are essential for maintaining the cellular redox environment and play important roles in regulating various cellular physiological functions. A fluorescence probe (compound **1-Cu**^**2+**^) for thiols based on coumarin carbohydrazide dinuclear copper complex was developed. Compound **1** was synthesized from the reaction of 7-(diethylamino)-2-oxo-2H-chromene-3-carbohydrazide with 4-tert-butyl-2,6- diformylphenol. Accordingly, the copper complex (compound **1-Cu**^**2+**^**)** was prepared by mixing compound **1** with 2 equivalents copper ions. Compound **1** had strong fluorescence while compound **1-Cu**^**2+**^ hardly possessed fluorescence owing to the quenching nature of paramagnetism Cu^2+^ to the fluorescence molecule excited state. However, the fluorescence intensity of compound **1-Cu**^**2+**^ was increased dramatically after the addition of thiol-containing amino acids, but not the other non-sulfhydryl amino acids. UV-vis absorption and fluorescence spectra indicated that compound **1-Cu**^**2+**^ had good selectivity and sensitivity for thiols such as glutathione in CH_3_CN:H_2_O (3:2, v/v) PBS solution. The fluorescence imaging experiments implied that compound **1-Cu**^**2+**^ has potential application in thiol-containing amino acids detection in living cells.

## Introduction

Biological thiols such as cysteine (Cys), homocysteine (Hcy), and glutathione (GSH) play essential roles in human physiology, and abnormal levels of these thiols are associated with a number of diseases [1–5]. For example, GSH, the most abundant intracellular nonprotein, serves many cellular functions, including maintenance of intracellular redox activities, xenobiotic metabolism, intracellular signal transduction, and gene regulation [6,7]. Particularly, GSH can keep the cysteine thiol group in proteins in the reduced state and protect the cells from oxidative stress by trapping free radicals that damage DNA and RNA [8]. The intracellular GSH concentration (1~10 mM) is substantially higher than extracellular levels (2 μM in plasma) [9,10], and the deficiency of which is involved in many diseases such as liver damage, leukocyte loss, cancer, AIDS and neurodegenerative diseases [11–13]. Therefore, the rapid, convenient, selective and sensitive detection of trace amounts of these thiols in biological and environmental samples consistently attracts a great deal of attention [14–16].

Among various analytical methods, fluorescence detection has proven to be one of the most convenient methods due to its simplicity, low cost, high sensitivity and great potential for intracellular bioimaging [3,11–13]. Currently, a number of organic reactions have been utilized to design fluorescence thiols probes [17–19], such as cyclization reactions between aldehydes and aminothiols [20–22], Michael addition reactions [23–25], cleavage reactions of 2,4-dinitrobenzenesulfonyl with thiols [26–28], nucleophilic substitution reactions [29–31], disulfide exchange reactions [32–34], and demetallation from Cu^2+^-complex [35]. The most molecular probes based on chemodosimetric reactions often suffer from the relatively longer incubation time ranging from 20 minutes to 1 hour or more. An alternate approach is to utilize the higher affinity of Cu(II) towards S-donor nucleophiles for designing molecular probes with fluorescence on response. Such reactions mostly occur within a millisecond time scale and thus are expected to be used in clinical detection [36]. This approach may improve the sensitivity due to thiols specific affinity of copper ions and also broaden the methodologies for designing various fluorescence probes [37–39].

Coumarins are one of the most widely used fluorophore for developing fluorescence probes. They are attractive starting materials for fluorogenic probes due to their high fluorescence intensity, excellent solubility, efficient cell permeation, and ease of preparation [40–42]. Herein, we report a new fluorescence probe compound **1-Cu**^**2+**^, which could be used for rapid, highly selective and sensitive detection of thiols. As a precursor, compound **1** was synthesized from the reaction of 7-(diethylamino)-2-oxo-2H-chromene-3-carbohydrazide (compound **3**) with 4-tert-butyl-2,6-diformylphenol (compound **2**). After coordinating with copper ions, the thiols fluorescence probe compound **1-Cu**^**2+**^, i.e., the coumarin carbohydrazide dinuclear copper complex, was prepared. Compound **1** had strong fluorescence while compound **1-Cu**^**2+**^ hardly possessed fluorescence. However, the fluorescence of compound **1-Cu**^**2+**^ was recovered after the addition of thiol-containing amino acids while non-sulfhydryl amino acids scarcely had impact on fluorescence probe. Thus, compound **1-Cu**^**2+**^ could be used to detect thiols such as glutathione, and has potential application in imaging of them in cells.

## Materials and Methods

^1^H NMR and ^13^C NMR spectra were measured on a Bruker Ascend^™^ 400 spectrometer with chemical shifts reported as ppm with TMS as internal standard. Mass spectrometric data were obtained with a Bruker Microtof-QIII spectrometry. UV-vis absorption spectra were recorded with Shimadzu UV2550 spectrophotometer. Fluorescence spectra were measured with Shimadzu RF-5301PC luminescence spectrometer. Excitation wavelengths for compound **1** and compound **1-Cu**^**2+**^ were both 445 nm. Both excitation and emission slit widths were 5 nm.

All the chemicals were of analytical grade and used as received. Stock solutions (2.0×10^−2^ M) of the perchlorate Cr^3+^, Ag^+^, Fe^3+^, K^+^, Na^+^, Mg^2+^, Pb^2+^, Ca^2+^, Hg^2+^, Mn^2+^, Cd^2+^, Fe^2+^, Zn^2+^, Ni^2+^, Co^2+^, Cu^2+^ and the amino acids plus GSH were prepared in aqueous solutions. Stock solutions of compound **1** and compound **1-Cu**^**2+**^ (10 μM) for spectral measurement were prepared in CH_3_CN:H_2_O (3:2 v/v) PBS solution. Stock solutions of compound **1** and compound **1-Cu**^**2+**^ for fluorescence imaging in cells were prepared in DMSO solution. Each time a 3 mL compound **1** or compound **1-Cu**^**2+**^ was filled in a quartz cell of 1 cm optical path length, and different stock solutions of metal ions or amino acids were added into the quartz cell gradually by using a micro-syringe.

**Fig 1:**
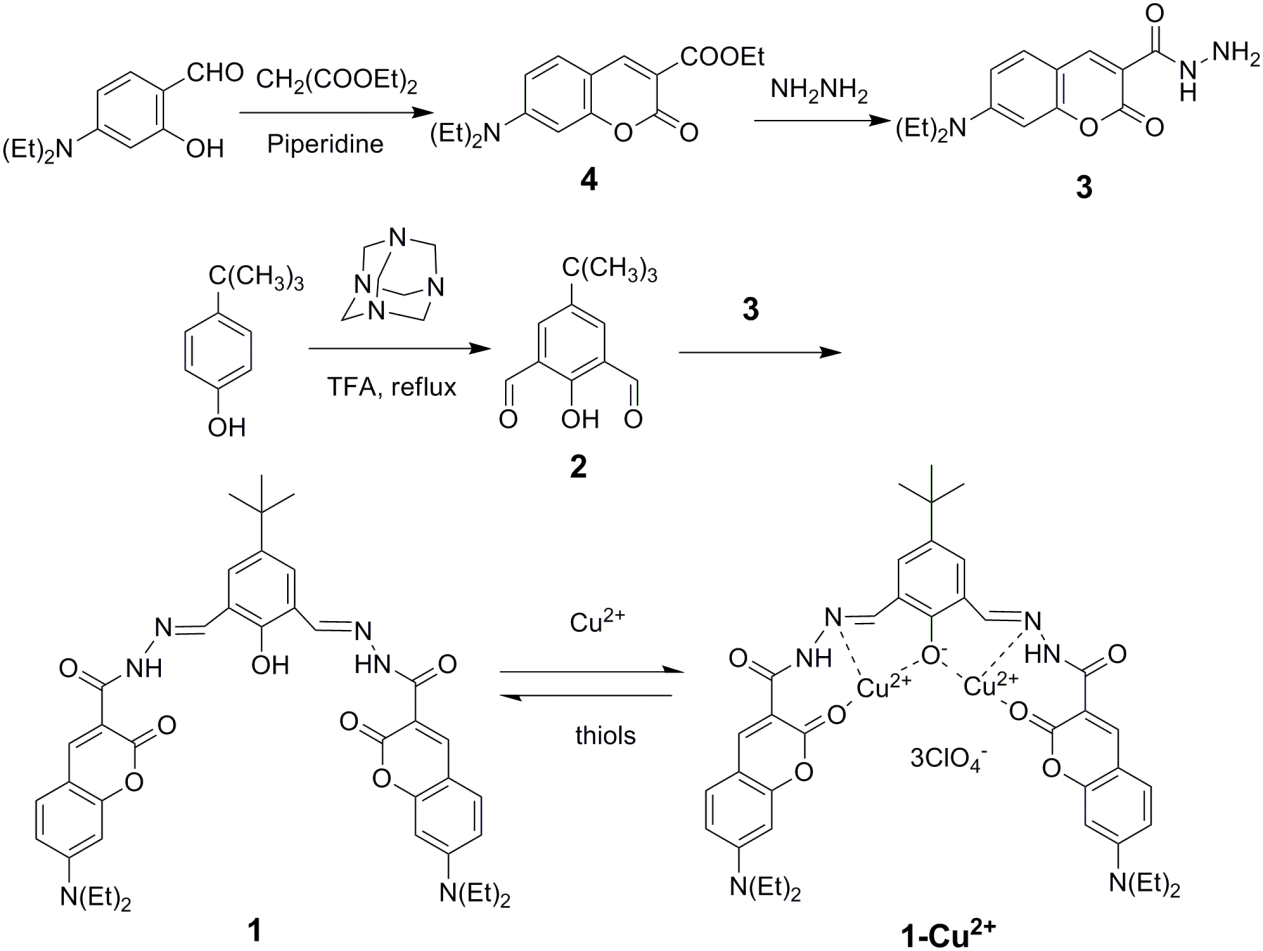
Synthesis procedures of the thiols probe (compound 1-Cu^2+^).

The synthesis procedures are shown in Fig 1. Ethyl 7-(diethylamino)-2-oxo-2H-chromene-3-carboxylate (compound **4**) and compound **3** were synthesized according to the literature [43,44]. 4-tert-butyl-2,6-diformylphenol was synthesized by the Duff reaction [45]. Compound **1** was synthesized conveniently from the reaction of compound **3** with compound **2** and characterized by ^1^H NMR, ^13^C NMR, and ESI-MS (S1A, S1B and S1C Fig). Compound **1-Cu**^**2+**^ was prepared by the reaction of compound **1** with Cu(ClO_4_)_2_ in MeOH and characterized by ESI-MS (S2A and S2B Fig) and IR spectra (S3A Fig).

## Results and Discussions

**Fig 2:**
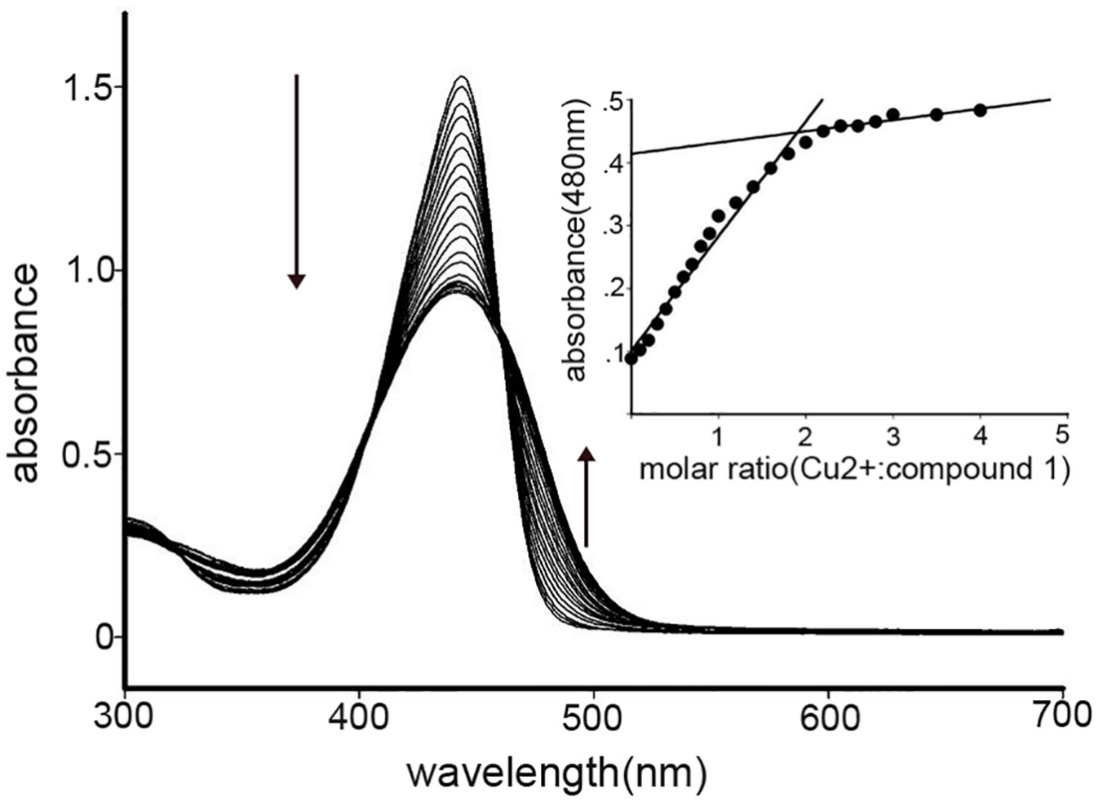
UV-vis titration of compound 1 (10 μmol/L) in CH_3_CN:H_2_O (3:2, v/v) PBS solution upon addition of Cu^2+^. Inset: UV-vis titration profile of compounds **1** upon addition of Cu^2+^, the absorption was recorded at 480 nm.

As shown in Fig 2, The UV-vis absorption spectra of compound **1** (10 μmol/L) in CH_3_CN:H_2_O (3:2, v/v) PBS solution exhibited a broad coumarin-based π-π* transition band around 445 nm (lgε = 5.18) [46]. To assess the chelating ability of compound **1,** the solution was titrated with copper ions. As shown in Fig 2, the band around 445 nm was decreased with increasing Cu(ClO_4_)_2_ and a new band centered around approximate 480 nm appeared, which was due to the coordination of compound **1** to Cu^2+^ [47]. The titration equilibrium was achieved upon addition of about 2 equivalents of copper ions. A distinct isosbestic point at 460 nm appeared indicated that a new species was formed. To determine the molar ratio of compound **1** to copper ions, the absorption spectra of varied concentrations of Cu(ClO_4_)_2_ in CH_3_CN:H_2_O (3:2, v/v) PBS solution at a fixed compound **1** concentration were recorded (Fig 2 insert). A 1:2 molar ratio between compound **1** and Cu(ClO_4_)_2_ was determined by Job’s method of continuous variations based on the absorbance changes at 480 nm. The UV-vis absorption spectra of compound **1** (10 μmol/L) to other metal ions in CH_3_CN:H_2_O (3:2, v/v) PBS solution are shown in S4 Fig. However, certain other metal ions such as Co^2+^ exhibited similar responses.

Furthermore, the solid form of compound **1-Cu**^**2+**^ was prepared in MeOH and characterized by ESI-MS and IR spectra. The evidence of complexation behavior of compound **1** (C_40_H_44_N_6_O_7_) with Cu^2+^ came directly from ESI-MS spectrum. As shown in S2A Fig, the predominated peak at m/z = 875.1904 corresponding to [(compound **1**)+2Cu^2+^+CH_3_OH-3H^+^]^+^ = C_41_H_45_Cu_2_N_6_O_8_^+^ was observed in ESI-MS spectrum. CH_3_OH molecules came from the mobile phase for dilution in ESI-MS spectra experiment and the loss of the three H^+^ came from one phenolic hydroxyl group and two amide groups. The experimental isotopic patterns (S2B Fig) fit well with the theoretical simulation result calculated by using the IsoPro 3.0 program (S2C Fig). Such results further indicated that the composition of compound **1-Cu**^**2+**^ is 1: 2 molar ratio between compound **1** and Cu^2+^, which was consistent with that of Job’s method.

IR spectrum of compound **1-Cu**^**2+**^ revealed the possible coordination model of compound **1** with copper ions (S3A Fig). As comparison, IR spectrum of compound **1** was also performed (S3B Fig). IR spectrum of compound **1** exhibited characteristic peaks of certain groups such as the carbonyl group of the coumarin unit (1689.64 cm^-1^) [48], the carbonyl group of -CO-NH- unit (1616.35 cm^-1^) and -C = N- group (1581.63 cm^-1^), etc [49]. After coordinated with copper ions, the characteristic amide carbonyl absorption of the coumarin moiety was shifted to 1701.22 cm^-1^ and the stretching band of the -C = N- group was shifted to 1589.34 cm^-1^. Nevertheless, the carbonyl group of -CO-NH- unit only shifted slightly (S3C Fig). It is assumed that both the carbonyl group of the coumarin unit and the -C = N- group participated in the coordination (Fig 1).

**Fig 3:**
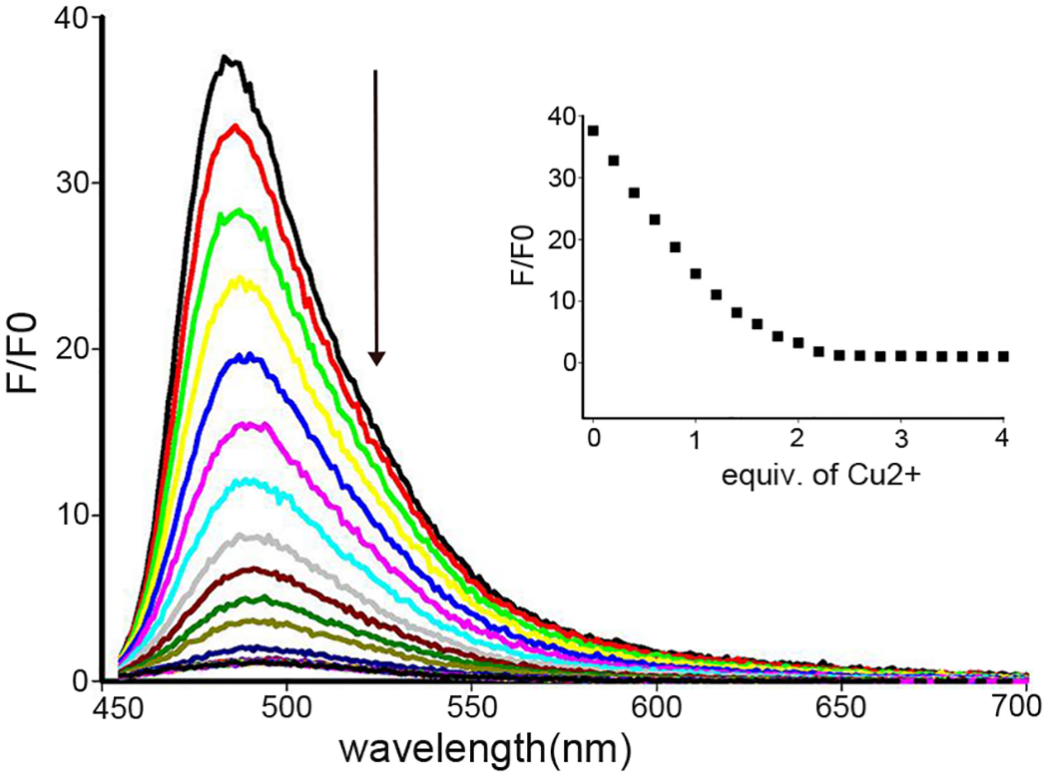
Fluorescence emission spectra of compound 1 (10 μmol/L) in CH_3_CN:H_2_O (3:2, v/v) PBS solution with successive addition of Cu^2+^. Insert: fluorescence titration profile at 483 nm upon the addition of Cu^2+^ (excited at 445 nm).

Fluorescence titration of compound **1** in CH_3_CN:H_2_O (3:2, v/v) PBS solution upon the addition of Cu^2+^ was also performed (Fig 3). When excited at 445 nm, compound **1** exhibited a strong fluorescence band at about 483 nm corresponding to the typical emission of the coumarin compound [46]. Upon the addition of increasing Cu^2+^ ions, the thiols probe (compound **1-Cu**^**2+**^) was prepared in situ, followed by the fluorescence intensity quenched significantly until the addition of about 2 equivalents of copper ions and then leveled off. The low luminescence intensity of compound **1-Cu**^**2+**^ is likely to result from the quenching effect by Cu^2+^ through a PET mechanism and/or a paramagnetic quenching mechanism [50]. The fluorescence responses of compound **1** (10 μmol/L) to various metal ions in CH_3_CN:H_2_O PBS (3:2, v/v) solutions were shown in S5 Fig. Nevertheless, certain other metal ions such as Co^2+^, Fe^2+^, Zn^2+^, exhibited similar quenching responses.

**Fig 4:**
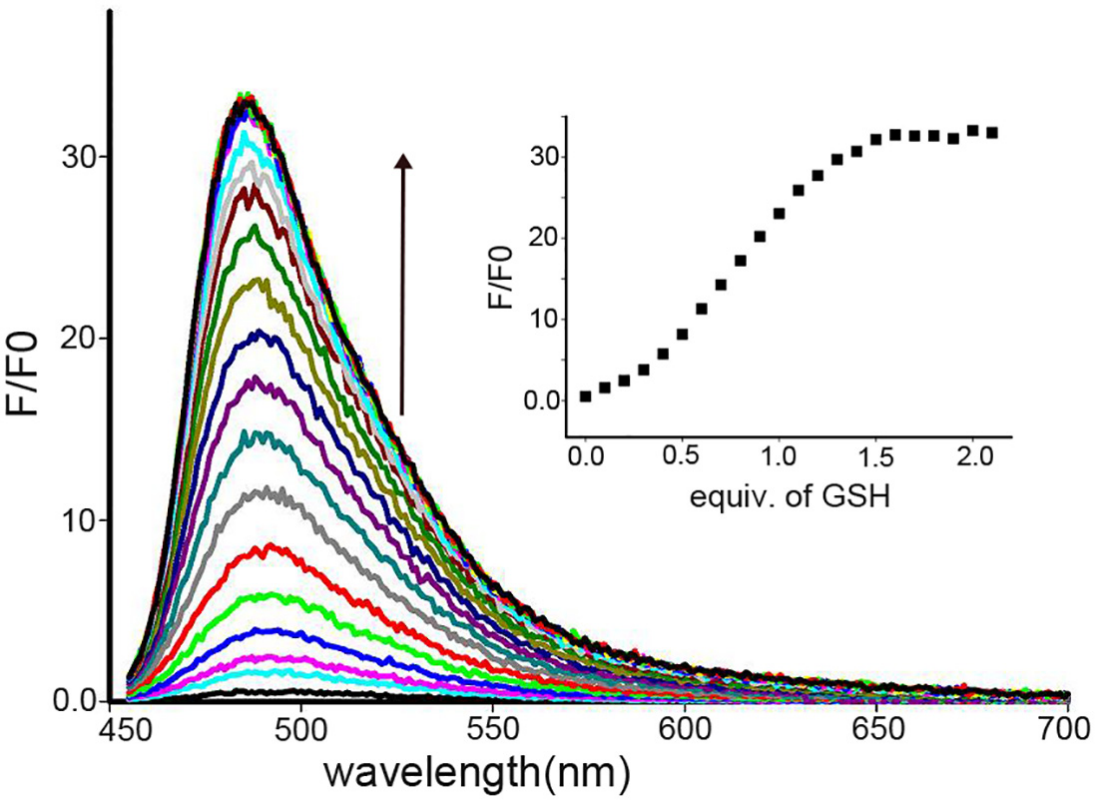
Fluorescence responses of compound 1-Cu^2+^ (10 μmol/L) in CH_3_CN:H_2_O (3:2, v/v) PBS solution upon the addition of increasing GSH. Insert: fluorescence titration profile at 483 nm upon the addition of GSH (excited at 445 nm).

However, after the succeeding addition of thiols such as GSH, the thiols probe released compound **1** owing to the demetallation, leading to the fluorescence recovery. As shown in Fig 4, the addition of GSH to compound **1-Cu**^**2+**^ (10 μmol/L) in CH_3_CN:H_2_O (3:2, v/v) PBS solution caused a dramatic immediate increase in emission intensity up to the maximum when about 1.7 equivalents GSH were added and then leveled off. Thus compound **1-Cu**^**2+**^ could be used to detect thiols with fluorescence on response.

**Fig 5:**
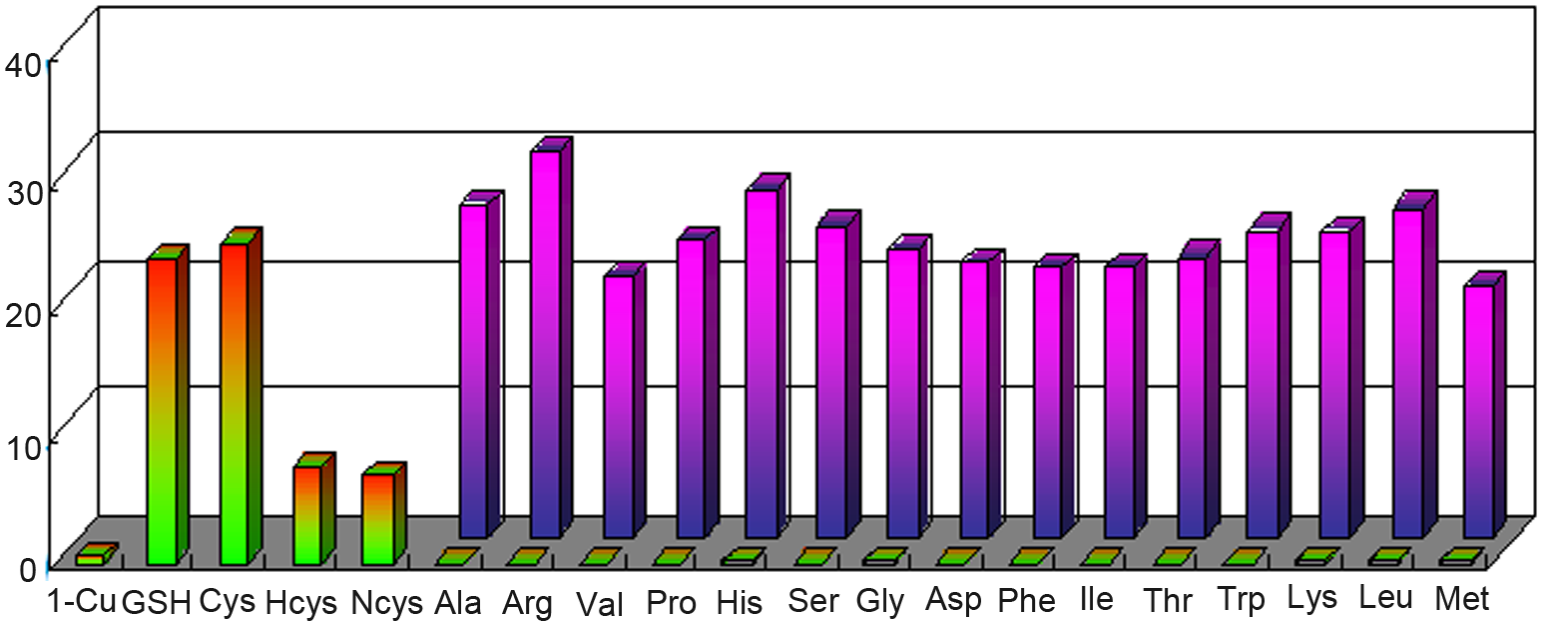
Fluorescence intensity (λ_em_ = 483 nm) of compound 1-Cu^2+^ to various amino acids: the first bars represent the fluorescence intensity upon addition of 4 equivalents of various amino acids; the second bars represent the fluorescence intensity after subsequent addition of 2 equivalents of GSH to the non-sulfhydryl amino acids solution, respectively.

To further explore the availability of compound **1-Cu**^**2+**^ as a highly selective probe for thiols, the fluorescence spectra of compound **1-Cu**^**2+**^ (10 μmol/L, CH_3_CN:H_2_O = 3:2, v/v) coexisting with the other amino acids that could probably affect the fluorescence were examined. As shown in Fig 5, the fluorescence intensity of the probe itself was very weak. When 2 equivalents thiol-containing amino acids such as L-cysteine, N-acetyl-cysteine, L-homocysteine acid and GSH were added respectively, the fluorescence intensity enhanced drastically. By comparison, when 4 equivalents various non-sulfhydryl amino acids such as L-tryptophan, L-glycine, L-lysine, L-histidine, L-glutamine, proline, methionine, leucine and isoleucine were added respectively, no obvious changes in the fluorescence spectra were observed. However, subsequent adding 2 equivalents GSH to the above non-sulfhydryl amino acids solutions gave rise to obvious increments of the fluorescence intensities, revealing that thiols had specific effects on the luminescence spectra. It should be noted that the fluorescence intensity was higher when 2 equivalents of GSH were added to compound **1-Cu**^**2+**^ solutions which have been added with 4 equivalents certain amino acids such as Ala, Arg, or His, in comparison to the direct addition of 2 equivalents of GSH to compound **1-Cu**^**2+**^ solutions, indicating that anti-interference in this system was slightly weak. In general, small organic fluorescence probes might be affected by the environment. In these experiments, the existence of certain amino acids might slightly affect the physical and chemical properties such as viscosity, polarity and pH value of the system, leading to fluorescence fluctuations in different amino acids. The above experiments indicated that compound **1-Cu**^**2+**^ had moderate selectivity to thiol-containing amino acids.

From a mechanistic viewpoint, the off-on fluorescence response of compound **1-Cu**^**2+**^ upon addition of GSH should be mainly attributed to the displacement effect. The direct evidence of the displacement mechanism came from ESI-MS spectra. Compound **1-Cu**^**2+**^ itself exhibited a dominant peak at m/z = 875.1904 in CH_3_CN:H_2_O (3:2, v/v) solution (S2A Fig), however, upon addition of 2 equivalents of GSH, this peak disappeared and a new peak at m/z = 721.3311 corresponding to compound **1** emerged (S6A and S6B Fig). Meanwhile, another new peak at m/z = 613.1584 corresponding to GSSG (oxidized glutathione) developed (S6A and S6C Fig). It could be speculated that the substitution reaction was accompanied by the redox reaction. On the one hand, Cu^2+^ in compound **1-Cu**^**2+**^ was taken away by glutathione. On the other hand, at the same time, Cu^2+^ was reduced to Cu^+^ and GSH was oxidized to GSSG. Such species might account for the fluorescence recovery: compound **1**, **Cu(I)-Ligand**, and even **Cu(I)-GSH** or **Cu(I)-GSSG**. To further explore the sources of the fluorescence recovery, some control experiments were carried out. To examine the fluorescence of compound **Cu(I)-Ligand**, univalent Cu(CH_3_CN)_4_ClO_4_ was used to prepare compound **Cu(I)-Ligand** in solution in situ. Upon addition of Cu(CH_3_CN)_4_ClO_4_ to compound **1**, the fluorescence of which was rapidly decreased (S7 Fig), implying that the fluorescence of compound **Cu(I)-Ligand** was extremely weak and should not be responsible for the reproduced fluorescence. Similar fluorescence experiments indicated that **Cu(I)-GSH** or **Cu(I)-GSSG** had no fluorescence (S8A and S8B Fig). Therefore, the fluorescence should in all probability derive from compound **1**.

To investigate the effect of anions on the probe, the fluorescence titration experiments of compound **1** (10 μmol/L) in CH_3_CN:H_2_O (3:2, v/v) PBS solution upon addition of increasing concentrations of other copper compounds such as Cu(NO_3_)_2_, Cu(OAc)_2_, CuCl_2_ and CuSO_4_, and successive addition of GSH were also performed, respectively. Compared with Cu(ClO_4_)_2_, the addition of the other copper compounds gave rise to similar responses in fluorescence titration spectra (S9A, S9B, S9C, S9D, S9E, S9F, S9G and S9H Fig).

**Fig 6:**
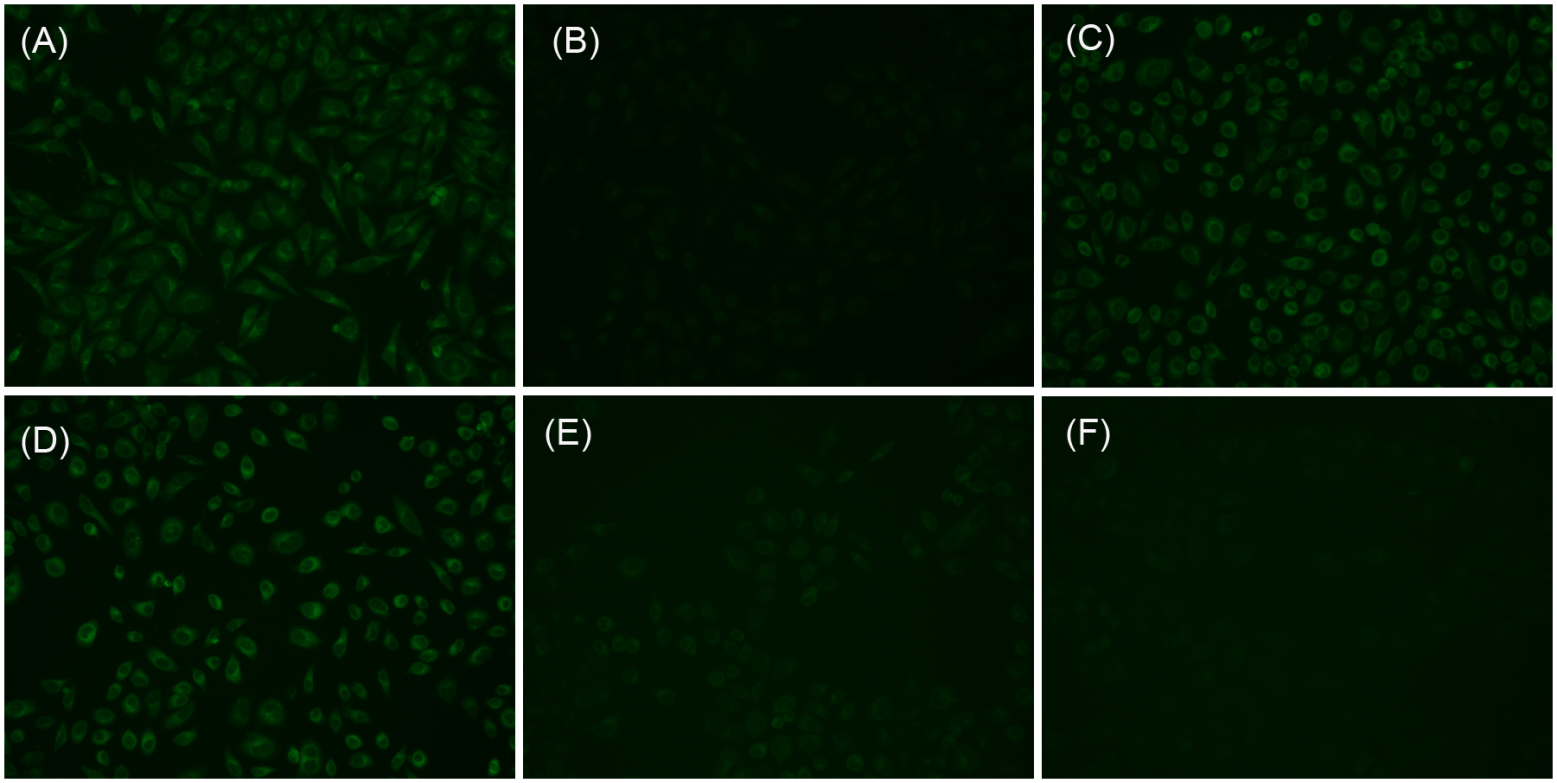
Fluorescence images of compound **1** (a); compound **1** and 3 equivalents of **Cu**^**2+**^ (b); compound **1**, 3 equivalents of **Cu**^**2+**^ and 6 equivalents of GSH (c); compound **1** as control (d); compound **1-Cu**^**2+**^ (e); and pretreated by erlotinib, compound **1-Cu**^**2+**^ (f) in SiHa cells.

The ability of biosensing molecules to selectively monitor guest species in living cells is of great importance for biological application [51]. The fluorescence imaging experiments of compound **1** and compound **1-Cu**^**2+**^ for GSH were carried out with cervical cancer SiHa cells. Firstly, the SiHa cells at exponential phase in 12-well plates were incubated with compounds **1** (2 μM) for 60 minutes at 37°C, then followed by PBS washing twice. The images were recorded by a Nikon Eclipse TE2000-S inverted fluorescence microscopy with a 20× objective lens (excited with blue light). The SiHa cells showed a clear green intracellular fluorescence (Fig 6A). To simulate the features of investigated compounds in vitro, to the wells three equivalents Cu^2+^ were added, and the fluorescence of the SiHa cells were dramatically quenched (Fig 6B). However, the cells regained fluorescence upon addition of six equivalents GSH after washing away Cu^2+^ (Fig 6C). The results prompted us to investigate the potential application of the compound **1-Cu**^**2+**^ in thiol detection in living cells. To test the hypothesis, the compound **1-Cu**^**2+**^ was used directly to stain the SiHa cells. In our preliminary experiment, compound **1-Cu**^**2+**^ could penetrate cellular lipid membranes. To confirm this viewpoint, other copper compounds such as Cu(NO_3_)_2_, Cu(OAc)_2_, CuCl_2_ and CuSO_4_ complexes were also prepared and applied in cell imaging (S10 Fig). Compared with Cu(ClO_4_)_2_ complexes, the other copper compounds gave rise to similar results in cell imaging, which indicated that copper complexes were able to penetrate cellular lipid membranes, instead of relying on the anions. Thus, compound **1-Cu**^**2+**^ could be directly used to stain the SiHa cells. One well was pretreated by erlotinib (an anticancer drug) for 1 hour and another well was not. The treatment of erlotinib was to create a redox stress environment, decreasing the intracellular GSH content, which has been revealed in many studies [52,53]. The images of the SiHa cells were shown in Fig 6E and 6F (Fig 6D as control). As expected, the difference in fluorescence intensity between the drug treated and without was observed, prompting that there was a difference in abundance of cellular thiol (amino acids). Therefore, the fluorescence imaging of compound **1-Cu**^**2+**^ may have potential application in imaging GSH (thiol containing amino acids) in living cells, yet more studies were required in future.

## Conclusions

In conclusion, a coumarin carbohydrazide dinuclear Schiff base compound (compound **1**) was synthesized from the reaction of 7-(diethylamino)-2-oxo-2H-chromene-3-carbohydrazide with 4-tert-butyl-2,6- diformylphenol. After compound **1** coordinated with copper ions, the thiols fluorescence probe (compound **1-Cu**^**2+**^) was prepared. The fluorescence spectra indicated that the probe had good selectivity and sensitivity for thiols such as GSH in CH_3_CN:H_2_O (3:2, v/v) solution. Particularly, the probe has potential application in imaging GSH in living cells.

## Supporting Information

S1 Fig Spectra of compound 1. ^1^H NMR of compound **1** in CDCl_3_ **(Figure A).** ^13^C NMR of compound **1** in CDCl_3_ **(Figure B).** ESI-MS of compound **1 (Figure C).**
(TIF)

S2 Fig ESI-MS spectra of compound 1-Cu^2+^. ESI-MS spectra of compound **1-Cu**^**2+**^ **(Figure A).** The experimental isotope distributions of the peak interested in ESI-MS spectra of compound **1-Cu**^**2+**^ **(Figure B).** The theoretical simulation result of the isotope distributions of the peak interested in ESI-MS spectra of compound **1-Cu**^**2+**^ according to the IsoPro 3.0 ESI-MS spectrum simulation program **(Figure C).**
(TIF)

S3 Fig IR spectra of compound 1-Cu^2+^ and compound 1. IR spectrum of compound **1-Cu**^**2+**^ **(Figure A).** IR spectrum of compound **1 (Figure B).** IR spectra contrast between compound **1** and **1-Cu**^**2+**^. Compound **1**: 1. 1689.64 cm^-1^; 2. 1616.35 cm^-1^; 3. 1581.63 cm^-1^. compound **1-Cu**^**2+**^: 1a. 1701.22 cm^-1^; 2a. 1618.28 cm^-1^; 3a. 1589.34 cm^-1^ **(Figure C).**
(TIF)

S4 Fig UV-vis absorption spectra of compound 1. UV-vis absorption spectra of compound **1** (10 μmol/L) to various metal ions in CH_3_CN:H_2_O (3:2, v/v) PBS solution.
(TIF)

S5 Fig Fluorescence responses of compound 1. Fluorescence responses of compound **1** (10 μmol/L) to various metal ions in CH_3_CN:H_2_O PBS (3:2, v/v) solution. The intensities were recorded at 483 nm with excitation at 445 nm.
(TIF)

S6 Fig ESI-MS spectra of compound 1-Cu^2+^ upon addition of 2 equivalents of GSH. ESI-MS spectrum of compound **1-Cu**^**2+**^ in CH_3_CN:H_2_O (3:2, v/v) solution upon addition of 2 equivalents of GSH **(Figure A).** The experimental isotope distributions of the peak at m/z = 721.3311 corresponding to the free ligand (compound **1**) in the ESI-MS spectrum of compound **1-Cu**^**2+**^ upon addition of 2 equivalents of GSH **(Figure B).** The experimental isotope distributions of the peak at m/z = 613.1584 corresponding to GSSG (oxidized glutathione) in the ESI-MS spectrum of compound **1-Cu**^**2+**^ upon addition of 2 equivalents of GSH **(Figure C).**
(TIF)

S7 Fig Fluorescence emission spectra of compound 1 with successive addition of Cu(CH_3_CN)_4_ClO_4_. Fluorescence emission spectra of compound **1** (10 μmol/L) in CH_3_CN:H_2_O (3:2, v/v) PBS solution with successive addition of Cu(CH_3_CN)_4_ClO_4_. Insert: fluorescence titration profile at 483 nm upon the addition of Cu(CH_3_CN)_4_ClO_4_ (excited at 445 nm).
(TIF)

S8 Fig Fluorescence emission spectra of Cu(I)-GSH and Cu(I)-GSSG. Fluorescence emission spectra of **Cu(I)-GSH** (10 μmol/L) in CH_3_CN:H_2_O (3:2, v/v) PBS solution (excited at 445 nm) **(Figure A).** Fluorescence emission spectra of **Cu(I)-GSSG** (10 μmol/L) in CH_3_CN:H_2_O (3:2, v/v) PBS solution (excited at 445 nm) **(Figure B)**.
(TIF)

S9 Fig Fluorescence titration experiments of compound 1 (10 μmol/L) in CH_3_CN:H_2_O (3:2, v/v) PBS solution upon addition of other copper compounds and successive addition of GSH. Fluorescence emission spectra of compound **1** upon addition of Cu(NO_3_)_2_ **(Figure A)** and successive addition of GSH **(Figure B)**. Fluorescence emission spectra of compound **1** upon addition of Cu(OAc)_2_ **(Figure C)** and successive addition of GSH **(Figure D)**. Fluorescence emission spectra of compound **1** upon addition of CuCl_2_ **(Figure E)** and successive addition of GSH **(Figure F)**. Fluorescence emission spectra of compound **1** upon addition of CuSO_4_ **(Figure G)** and successive addition of GSH **(Figure H)** Inserts were their corresponding fluorescence titration profiles.
(TIF)

S10 Fig Fluorescence images of copper compounds. Fluorescence images of compound **1**-Cu(ClO_4_)_2_ **(Figure A),** compound **1**-Cu(NO_3_)_2_ **(Figure B),** compound **1**-Cu(OAc)_2_ **(Figure C),** compound **1**-CuCl_2_ **(Figure D),** compound **1**-CuSO_4_ **(Figure E)** and their corresponding bright field **(Figure a-e)** in SiHa cells.
(TIF)
