# Supplementary figures and images for: The Synthesis of a Coumarin Carbohydrazide Dinuclear Copper Complex Based Fluorescence Probe and Its Detection of Thiols
Source: PLoS One. 2016 Feb 12;11(2):e0148026. doi: 10.1371/journal.pone.0148026 (PMC4752479; doi:10.1371/journal.pone.0148026)

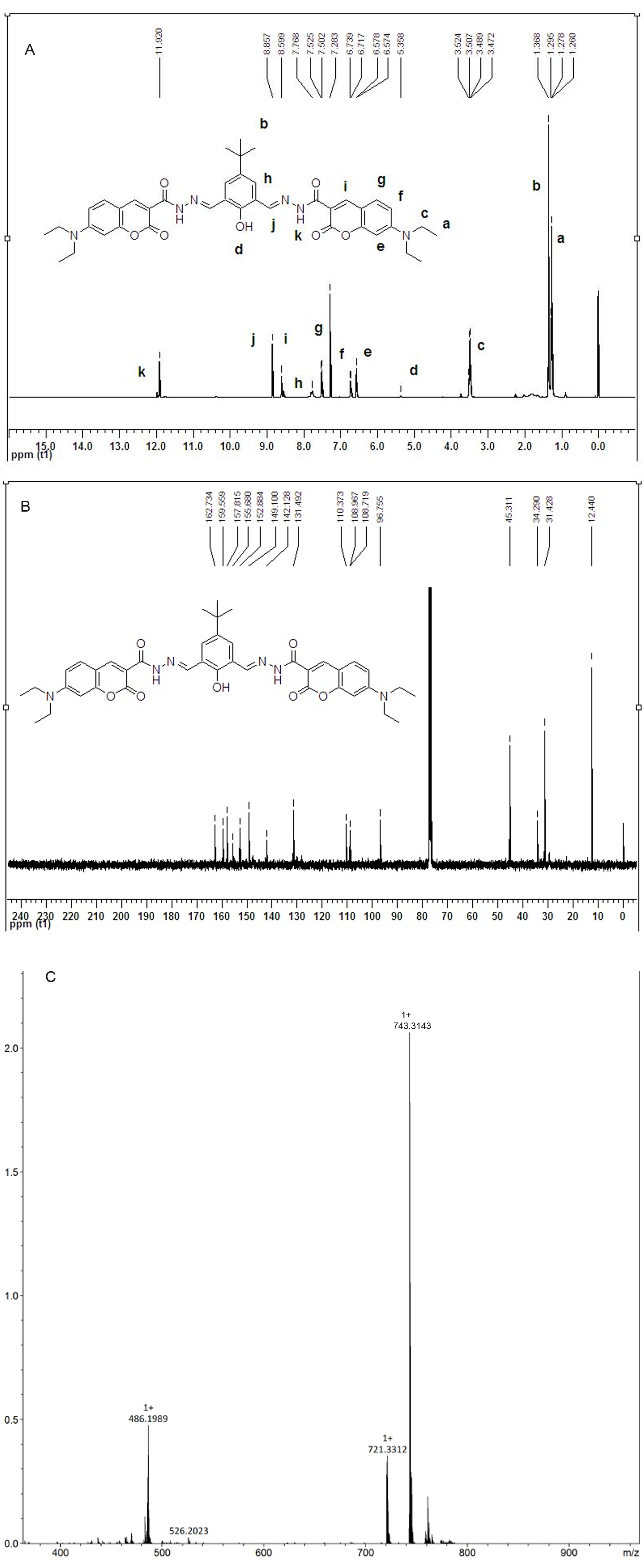

Supplement: S1 Fig — 1H NMR of compound 1 in CDCl3 (Figure A). 13C NMR of compound 1 in CDCl3 (Figure B). ESI-MS of compound 1 (Figure C). (TIF) [file pone.0148026.s001.tif]

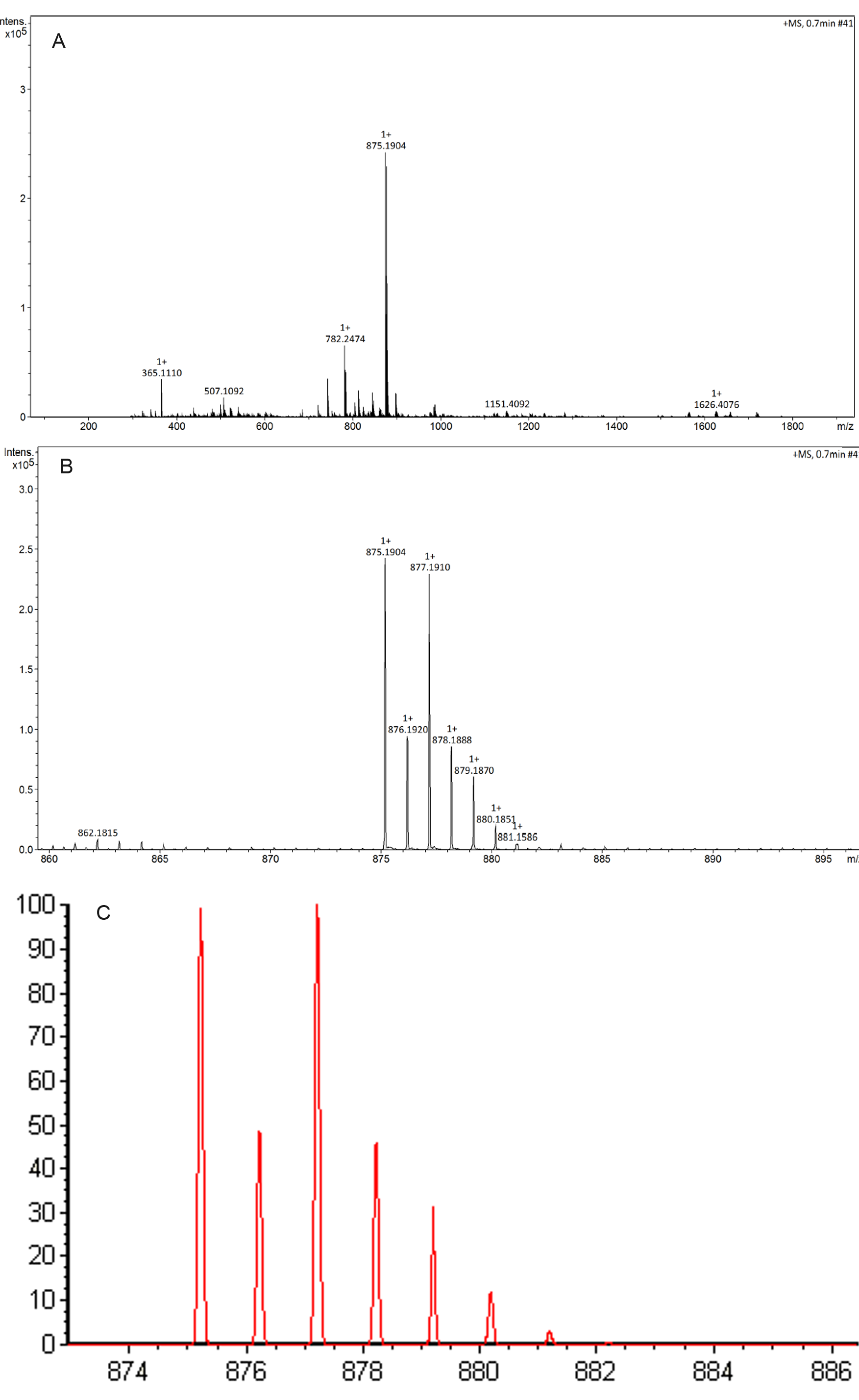

Supplement: S2 Fig — ESI-MS spectra of compound 1-Cu2+ (Figure A). The experimental isotope distributions of the peak interested in ESI-MS spectra of compound 1-Cu2+ (Figure B). The theoretical simulation result of the isotope distributions of the peak interested in ESI-MS spectra of compound 1-Cu2+ according to the IsoPro 3.0 ESI-MS spectrum simulation program (Figure C). (TIF) [file pone.0148026.s002.tif]

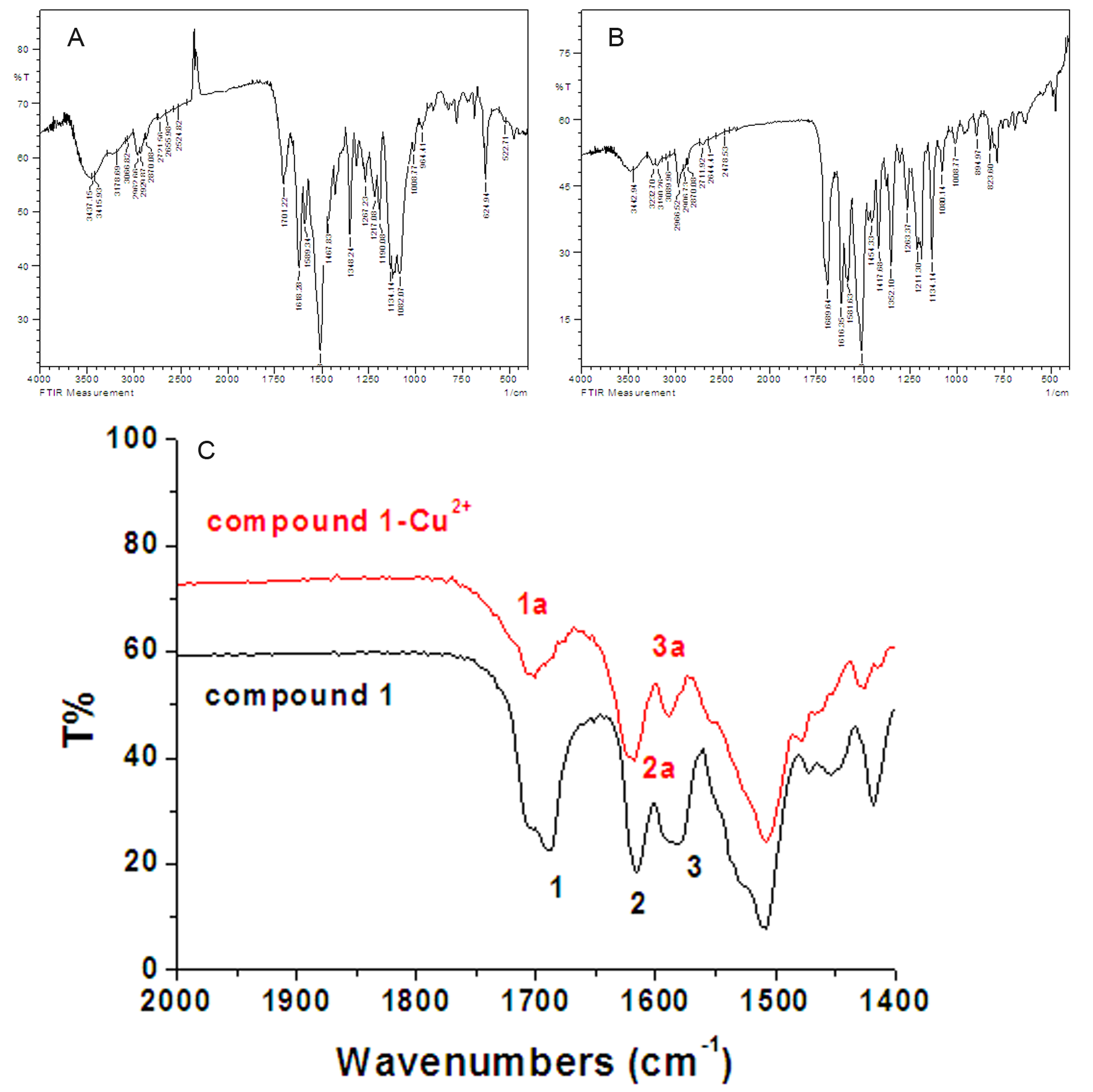

Supplement: S3 Fig — IR spectrum of compound 1-Cu2+ (Figure A). IR spectrum of compound 1 (Figure B). IR spectra contrast between compound 1 and 1-Cu2+. Compound 1: 1. 1689.64 cm-1; 2. 1616.35 cm-1; 3. 1581.63 cm-1. compound 1-Cu2+: 1a. 1701.22 cm-1; 2a. 1618.28 cm-1; 3a. 1589.34 cm-1 (Figure C). (TIF) [file pone.0148026.s003.tif]

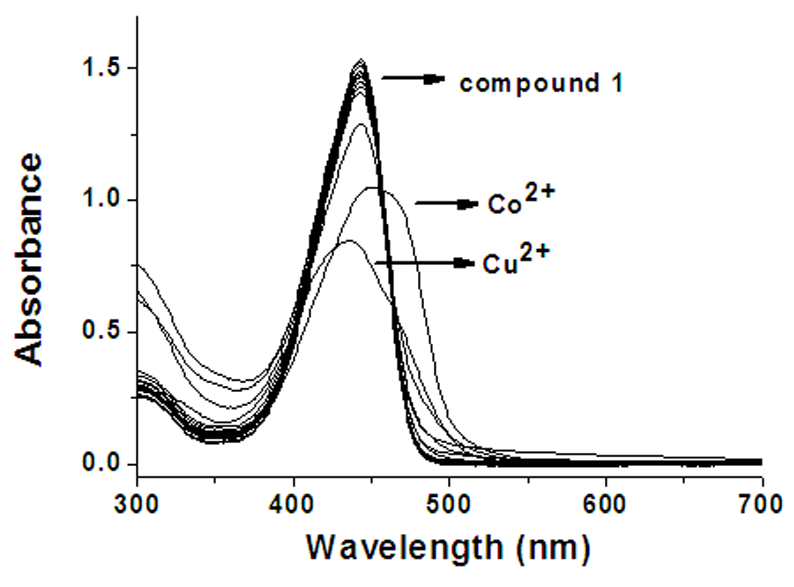

Supplement: S4 Fig — UV-vis absorption spectra of compound 1 (10 μmol/L) to various metal ions in CH3CN:H2O (3:2, v/v) PBS solution. (TIF) [file pone.0148026.s004.tif]

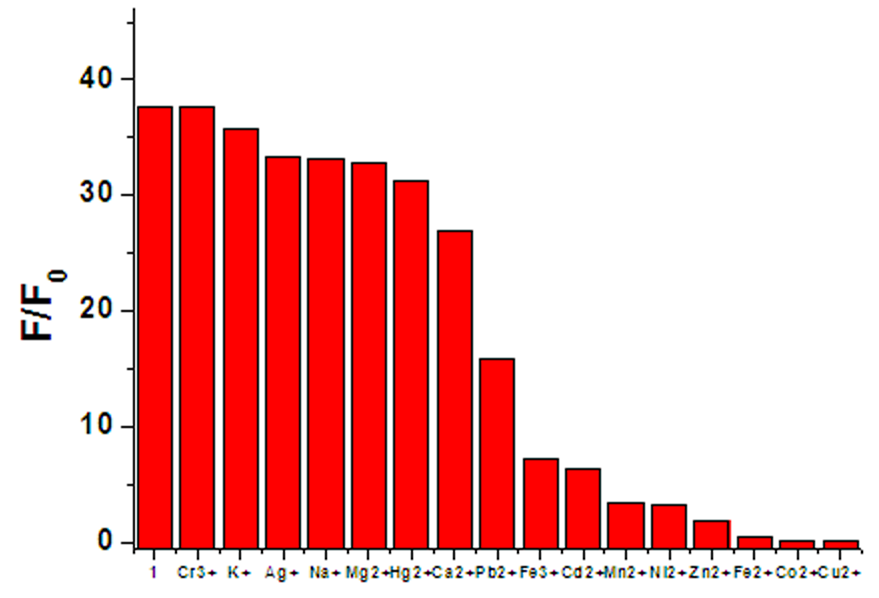

Supplement: S5 Fig — Fluorescence responses of compound 1 (10 μmol/L) to various metal ions in CH3CN:H2O PBS (3:2, v/v) solution. The intensities were recorded at 483 nm with excitation at 445 nm. (TIF) [file pone.0148026.s005.tif]

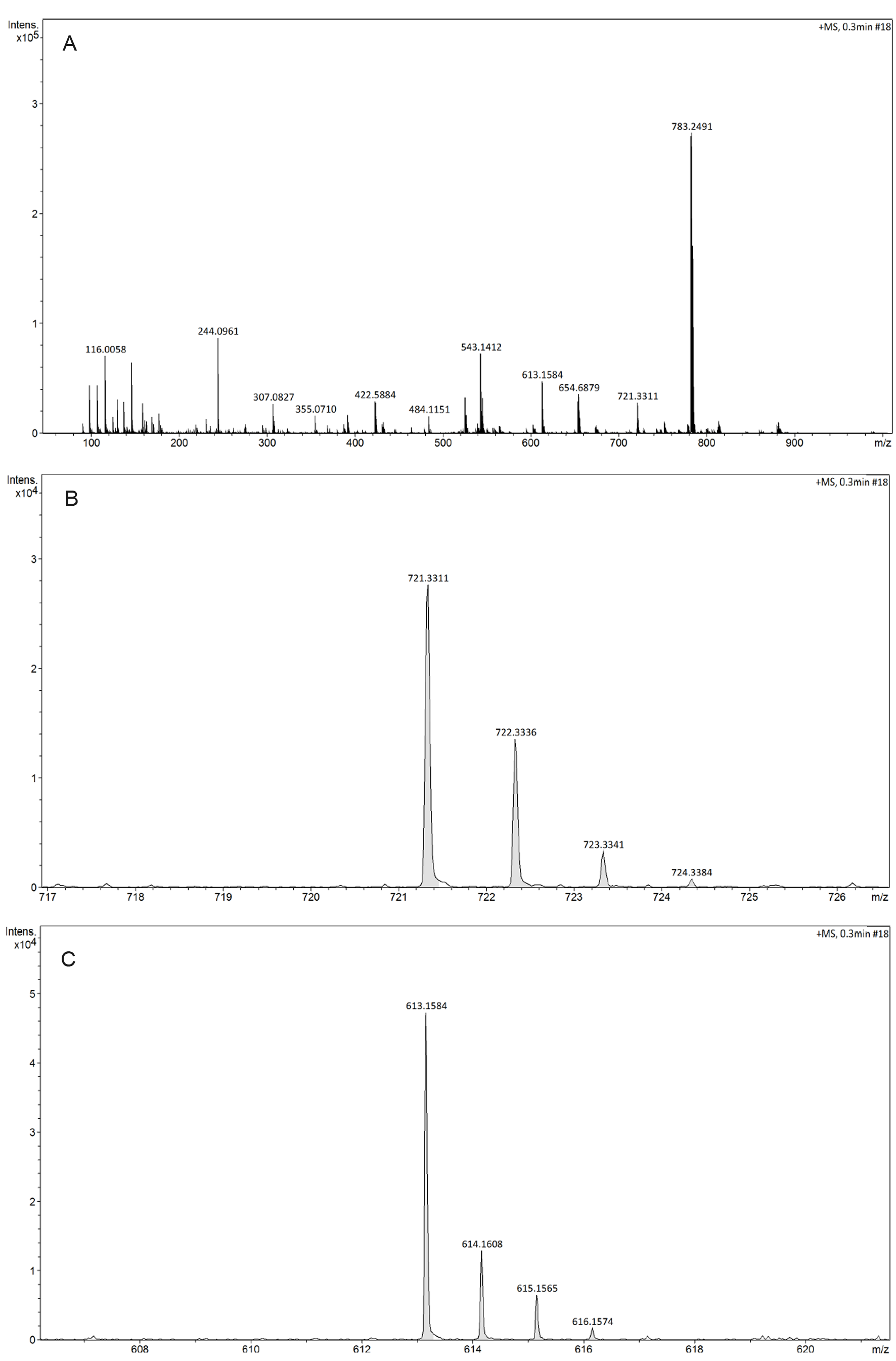

Supplement: S6 Fig — ESI-MS spectrum of compound 1-Cu2+ in CH3CN:H2O (3:2, v/v) solution upon addition of 2 equivalents of GSH (Figure A). The experimental isotope distributions of the peak at m/z = 721.3311 corresponding to the free ligand (compound 1) in the ESI-MS spectrum of compound 1-Cu2+ upon addition of 2 equivalents of GSH (Figure B). The experimental isotope distributions of the peak at m/z = 613.1584 corresponding to GSSG (oxidized glutathione) in the ESI-MS spectrum of compound 1-Cu2+ upon addition of 2 equivalents of GSH (Figure C). (TIF) [file pone.0148026.s006.tif]

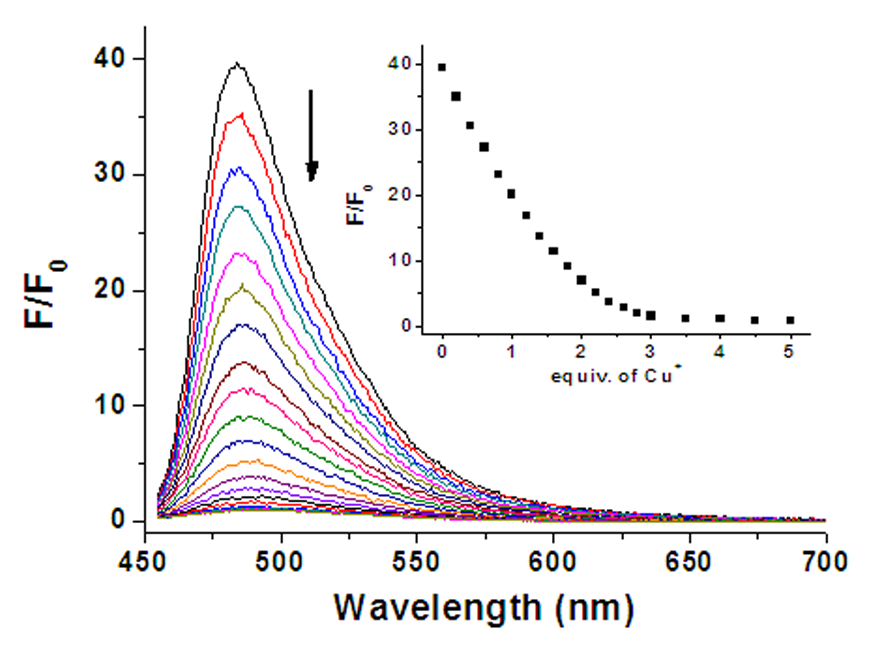

Supplement: S7 Fig — Fluorescence emission spectra of compound 1 (10 μmol/L) in CH3CN:H2O (3:2, v/v) PBS solution with successive addition of Cu(CH3CN)4ClO4. Insert: fluorescence titration profile at 483 nm upon the addition of Cu(CH3CN)4ClO4 (excited at 445 nm). (TIF) [file pone.0148026.s007.tif]

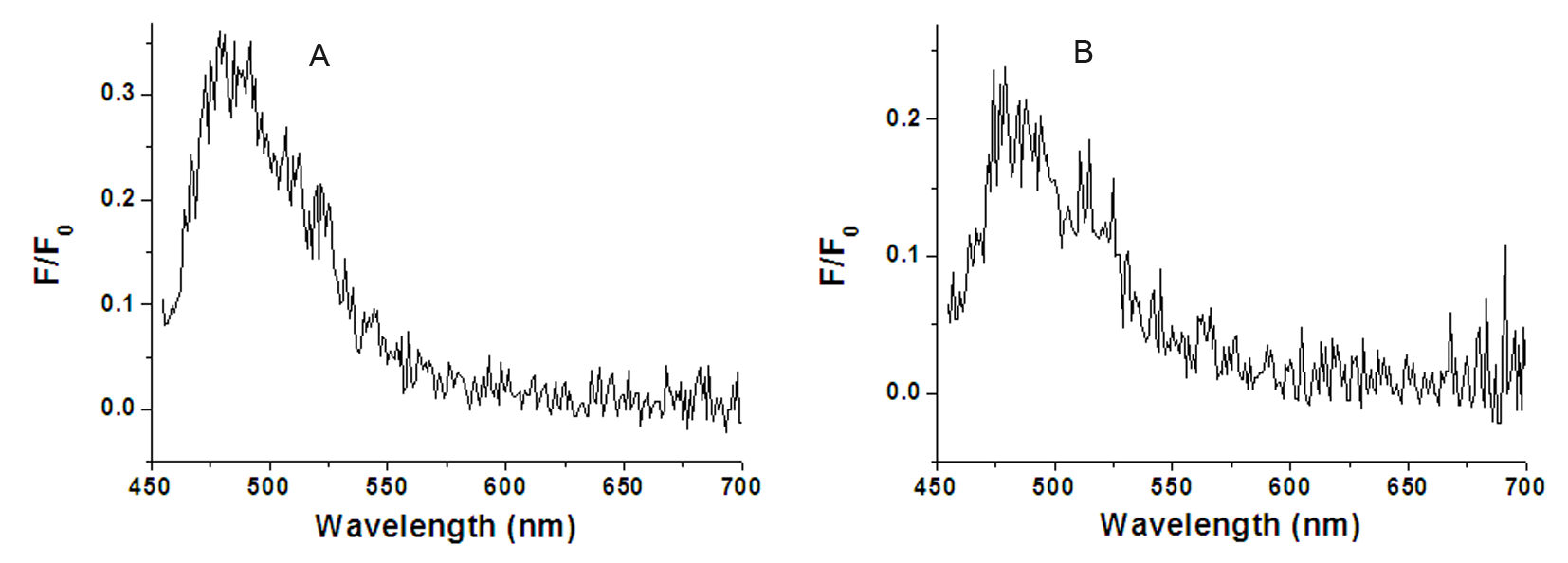

Supplement: S8 Fig — Fluorescence emission spectra of Cu(I)-GSH (10 μmol/L) in CH3CN:H2O (3:2, v/v) PBS solution (excited at 445 nm) (Figure A). Fluorescence emission spectra of Cu(I)-GSSG (10 μmol/L) in CH3CN:H2O (3:2, v/v) PBS solution (excited at 445 nm) (Figure B). (TIF) [file pone.0148026.s008.tif]

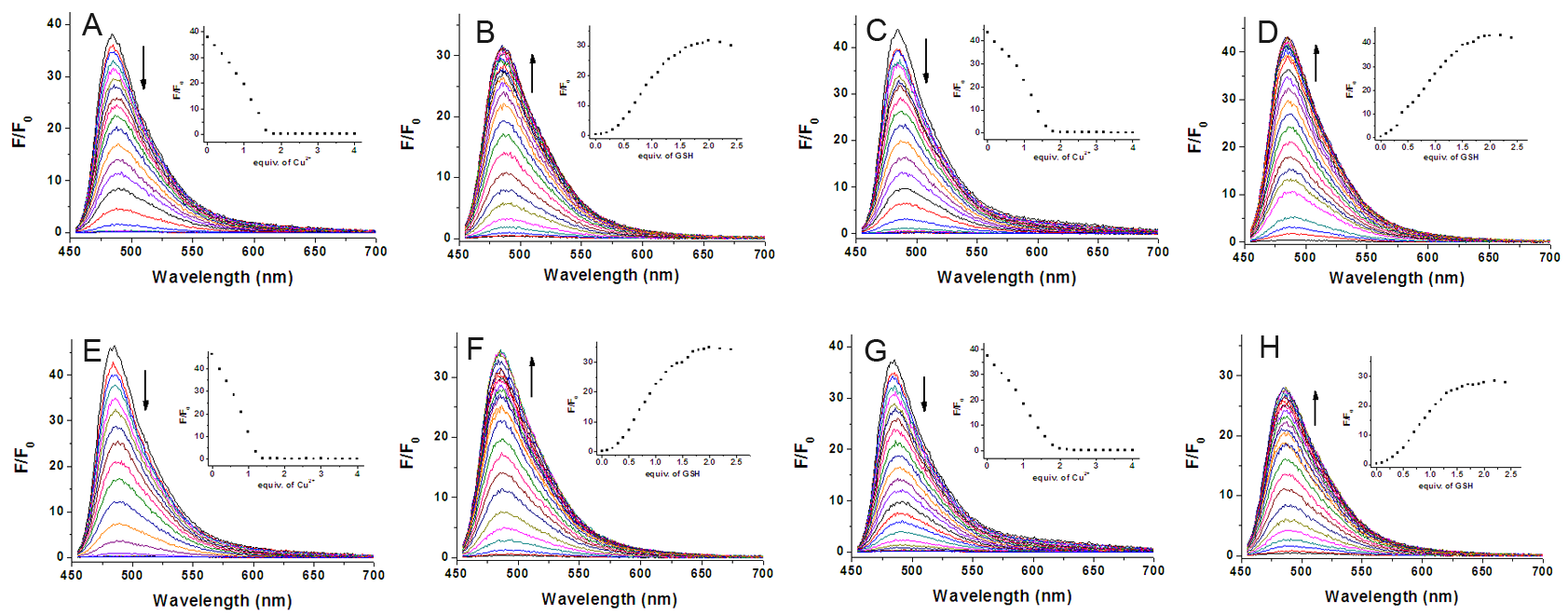

Supplement: S9 Fig — Fluorescence emission spectra of compound 1 upon addition of Cu(NO3)2 (Figure A) and successive addition of GSH (Figure B). Fluorescence emission spectra of compound 1 upon addition of Cu(OAc)2 (Figure C) and successive addition of GSH (Figure D). Fluorescence emission spectra of compound 1 upon addition of CuCl2 (Figure E) and successive addition of GSH (Figure F). Fluorescence emission spectra of compound 1 upon addition of CuSO4 (Figure G) and successive addition of GSH (Figure H) Inserts were their corresponding fluorescence titration profiles. (TIF) [file pone.0148026.s009.tif]

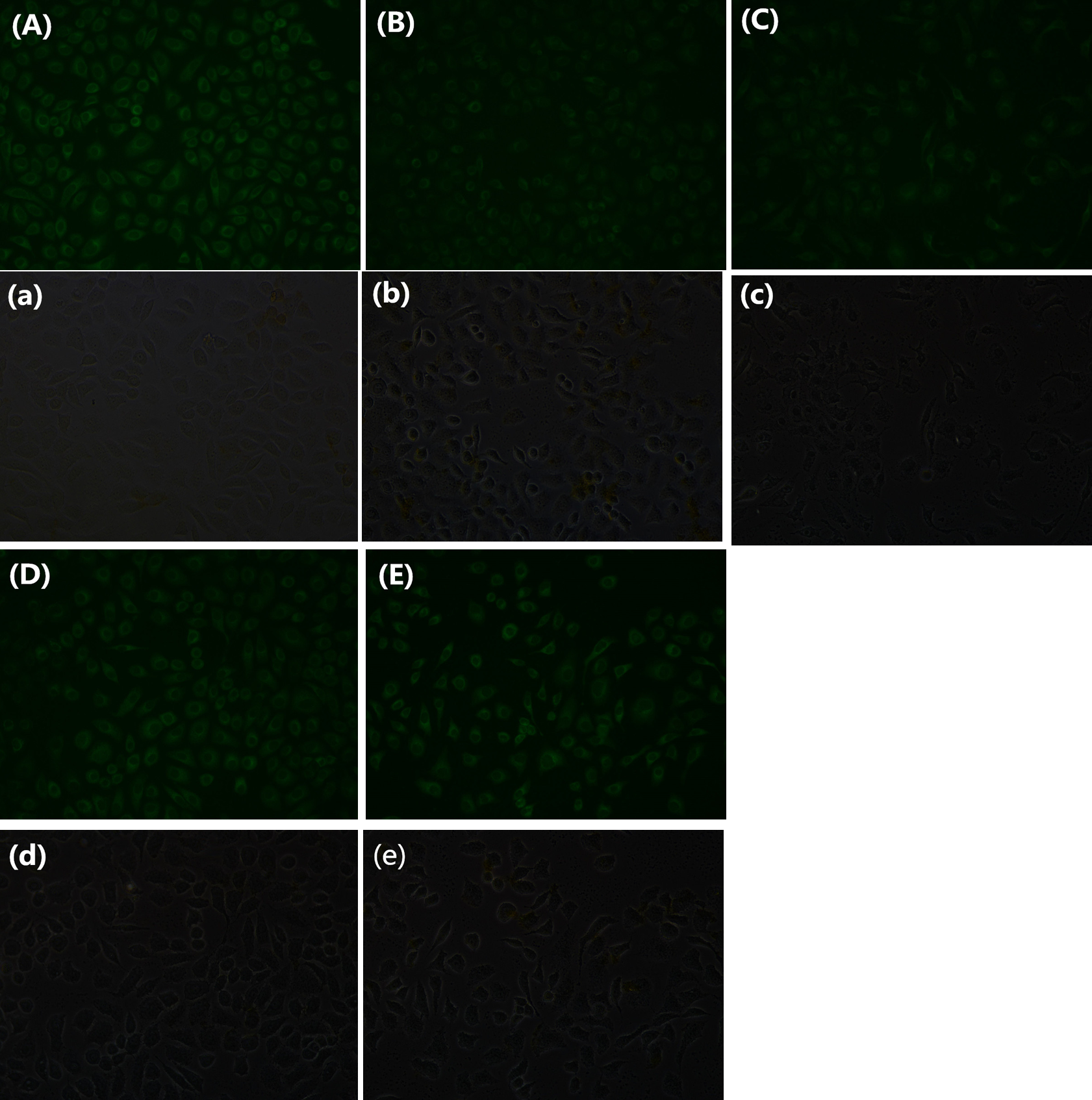

Supplement: S10 Fig — Fluorescence images of compound 1-Cu(ClO4)2 (Figure A), compound 1-Cu(NO3)2 (Figure B), compound 1-Cu(OAc)2 (Figure C), compound 1-CuCl2 (Figure D), compound 1-CuSO4 (Figure E) and their corresponding bright field (Figure a-e) in SiHa cells. (TIF) [file pone.0148026.s010.tif]
